# A New Method for Revision of Encapsulated Blebs after Trabeculectomy: Combination of Standard Bleb Needling with Transconjunctival Scleral Flap Sutures Prevents Early Postoperative Hypotony

**DOI:** 10.1371/journal.pone.0157320

**Published:** 2016-06-17

**Authors:** Panagiotis Laspas, Philipp David Culmann, Franz Hermann Grus, Verena Prokosch-Willing, Alicia Poplawksi, Norbert Pfeiffer, Esther Maria Hoffmann

**Affiliations:** Department of Ophthalmology, University Medical Center, Johannes Gutenberg- University Mainz, Mainz, Germany; Bascom Palmer Eye Institute, University of Miami School of Medicine;, UNITED STATES

## Abstract

**Purpose:**

A simple needling procedure is the standard method for restoring the function of an encapsulated bleb after trabeculectomy. However, postoperative hypotony represents a possible hazard. This study describes a new surgical approach for treating encapsulated blebs with reduced risk of early postoperative hypotony: bleb needling combined with transconjunctival sutures tightening the scleral flap directly.

**Methods:**

The study included two groups of 23 patients with failing bleb following trabeculectomy: “Group 1” underwent simple needling revision of the filtering bleb and served as a control group, while “Group 2” received needling revision with additional transconjunctival scleral flap sutures, if intraoperatively the intraocular pressure was estimated to be very low. Intraocular pressure (IOP), postoperative management and complications were analyzed over a follow-up period of 4 weeks postoperatively. Results were compared using t-test or Mann-Whitney U-tests.

**Results:**

Adverse effects occurred with a higher frequency after sole needling of the bleb (5 cases of choroidal effusion and 1 case of choroidal hemorrhage) than after the combined method with additional scleral sutures (1 case of choroidal effusion). The IOP on the first postoperative day was significantly lower in group 1, with 9.43 ± 9.01 mm Hg vs. 16.43 ± 8.35 mm Hg in group 2 (P = 0.01). Ten patients with ocular hypotony (IOD of 5 mmHg or lower) were found in group 1 and only two in group 2. One week and one month after surgery the intraocular pressure was similar in both groups (P>0.05).

**Conclusions:**

This new needling technique with additional transconjunctival scleral flap sutures appears to reduce postoperative hypotony, and may thus protect from further complications, such as subchoroidal hemorrhage.

## Introduction

In order to prevent visual impairment in glaucoma, a reduction of intraocular pressure (IOP) through medication or surgery is mandatory. In cases with uncontrolled IOP despite medication, trabeculectomy remains the gold standard among surgical procedures that offer an adequate IOP reduction [1]. Although the introduction of antimetabolites as an adjunctive to trabeculectomy has improved the long–term outcome significantly [2–4], encapsulation of the filtration bleb with consecutive outflow disturbance and an increase in IOP remains the most common postoperative problem, occurring in around 13% of cases [5, 6].

A common therapeutic option for an encapsulated bleb is transconjunctival needling of the bleb, a minimal invasive technique, which opens the scarring tissue and can restore filtration [7–9]. However, a major complication of needling—with an incidence reported between 15–30%—is postoperative hypotony [10, 11], especially if the scleral flap has to be lifted during the needling procedure. Typical signs of hypotony are flattening of the anterior chamber, overfiltration and choroidal effusion. One major complication is vision-threatening choroidal hemorrhage. Late ocular hypotony may cause maculopathy, chorioretinal folds of the posterior pole, tortuosity of the retinal vessels and visual loss [12, 13].

Conservative measures for the management of early ocular hypotony include the application of bandage contact lenses, pressure patching, or the use of acetazolamide to reduce the aqueous outflow [14]. If these are unsuccessful, surgical approaches such as the injection of autologous blood into the bleb in order to initiate scarring [15], the reopening of the conjunctiva to re-suture the scleral flap [16, 17], or supporting the flap by a donor patch [18], have been advocated. These methods may initiate a rapid raise of IOP, but on the other hand often lead to excessive bleb scarring and thereby possibly failure of surgery. An elegant approach, which has been shown to be successful to prevent hypotony, is the placement of transconjunctival scleral flap sutures. These have the potential to preserve the flap, raise IOP rapidly, and allow postoperatively adequate IOP regulation by later elective removal of these sutures [19, 20].

In order to reduce the frequency of acute ocular hypotony and to prevent its possibly disastrous complications, we developed a technique of combining the needling procedure with additional transconjuctival scleral flap sutures to avoid overfiltration.

In this retrospective study we report the results of this surgical approach on encapsulated blebs compared with results of patients who underwent a simple needling procedure. Outcome measures were number of cases of hypotony, postoperative IOP, and complications.

## Methods and Patients

The study was conducted according to the recommendations of the Declaration of Helsinki, and was approved through the local ethics committee of Rhineland-Palatinate. An interinstitutional, retrospective, interventional case series with 46 patients treated at the Department of Ophthalmology, Mainz University Medical Center between 2009 and 2014 was evaluated. All patients had a non-functional encapsulated bleb following previous trabeculectomy with elevated IOP, and bleb revision was indicated. The condition "elevated IOP" was individualized for each case, according to the stage of the disease and the targeted IOP. Patients were informed about the surgical procedure (necessity, prospects, risks) and gave signed consent. As the retrospective investigation of the results occurred years after the surgical procedure, separate consent from the patients for the use of their medical information in research could not be given.

The study included two groups of 23 patients: “Group 1” underwent simple needling revision of the filtering bleb, while “Group 2” received needling revision with additional transconjunctival scleral flap sutures. Surgery took place microscopically under retrobulbar or general anesthesia, taking into consideration the status of the fellow eye, the safety of the procedure, and the patient’s wishes.

After disinfection with 1% povidone-iodine solution (Braun, Melsungen, Germany) and intended depression of gaze, the conjunctiva was penetrated with a 14 gauge needle at a considerable distance from the scleral flap, in order to avoid postoperative leakage of aqueous humor. Subconjunctival scar tissue was incised around the scleral flap and along the encapsulation with the needle, in order to reestablish a subconjunctival space for the filtrated aqueous humor.

In the case of a non satisfactory filtration after this step, the needle was further advanced towards the scleral flap, episcleral fibrotic tissue was cut, the margins of the scleral flaps were reopened, and the needle was advanced under the flap, through the trabeculectomy opening, until its tip was visible in the anterior chamber. Generally, if the flap is lifted, in most cases the anterior chamber flattens and the IOP becomes very low. When we had such cases, one or more sutures (10–0 nylon with cutting needle, Type CU-8, Circle: Bi-Curve; Alcon, Freiburg, Switzerland) were placed through the conjunctiva and the scleral flap in order to tighten the flap and to restore IOP and deepen the anterior chamber.

The sutures were applied through the conjunctiva and the scleral flap, which was readapted to the adjacent sclera, and then emerged once more through the conjunctiva. One to three sutures, depending on the surgeon’s estimation of the IOP were placed and knotted tightly over the conjunctiva. In the following days, the sutures cut through the conjunctiva, penetrate it without injury, and eventually reach the subconjunctival space, where they remain in. This causes a relative relaxation of the elastic suture with consecutive relaxation also of the tension applied to the scleral flap, often followed by a moderate reduction of the IOP. In the case of a postoperative persistently high IOP, these sutures can be removed under topical anesthesia, either with fine surgical scissors and forceps, if the suture is reachable over the conjunctiva, or by argon laser, if the suture has cut into the subconjunctival space.

After surgery, application of subconunctival injections of 5 Fluorouracil (5-FU) was performed over 5–7 days in both groups in order to avoid another bleb encapsulation. The decision for a 5-FU injection was made taking into consideration morphologic and functional characteristics of the bleb and their development over time. Thick, intransparent conjunctiva with corkscrew vessels, no adequate filtration, and further signs of fibrosis, such as flat bleb, missing microcysts, and increased mobility of the conjunctiva versus the underlying tenon were typical indicators for the necessity of antimetabolites.

The needling group (Group 1) included 12 male and 11 female patients, whose average age was 72.78 ± 7.38 years. All patients were Caucasians. One patient had a positive family history of glaucoma. 15 patients had primary open-angle glaucoma, four pseudoexfoliative glaucoma, one patient pigment dispersion glaucoma, one patient juvenile glaucoma, one patient normal-tension glaucoma, and one patient secondary glaucoma (Tables 1 and 2).

**Table 1:** Demographics of the study groups and distribution of the different types of glaucoma in the study.

	Needling Patients (n)	Needling + Sutures Patients (n)
**Demographics**		
Age	72.78 ± 7.38	72.52 ± 6.96
Male/Female	12/11	11/12
Caucasians/Africans	23/0	23/0
**Type of Glaucoma**		
Open angle	15 (65.2%)	13 (56.5%)
Pseudoexfoliation	4 (17.4%)	7 (30.4%)
Normal pressure	1 (4.3%)	2 (8.7%)
Pigmentdispersion	1 (4.3%)	1 (4.3%)
Juvenile	1 (4.3%)	0
Secondary	1 (4.3%)	0

**Table 2:** Mean of intraocular pressure (IOP) of the study groups at specific time points until one month after bleb revision.

IOP (mmHg)	Needling Group	Needling + Sutures	P Value
before Revision	22.96 ± 7.57	21.09 ±4.37	0.59
Day 1 after Revision	9.43 ± 9.05	16.43± 8.35	**0.001**
Day 7	10.81 ± 8.03	11 ± 6.5	0.61
Month 1	13.58 ± 7.24	13.4 ± 4.93	0.9

In the needling plus transconjunctival scleral flap sutures group (Group 2) 11 male and 12 female patients with an average age of 72.52 ± 6.96 years were included. All patients were Caucasians. 13 patients suffered from open-angle glaucoma, 7 from pseudoexfoliative glaucoma, two from normal-tension glaucoma, and one from pigment dispersion glaucoma (Table 1).

All patients’ data (medical history, ocular parameters) were collected preoperatively. All bleb surgeries were performed at the Department of Ophthalmology of the University Medical Center in Mainz by the same surgeon (NP). Patients remained hospitalized for about one week and received daily slit-lamp and fundus examinations. An examination on an outpatient basis took place at one month after surgery.

Our major interest in this study was the incidence of ocular hypotony and its complications. Hypotony was defined as the status of an IOP of 5 mmHg or lower, the same measurement used in previous studies [10, 11]. We performed descriptive statistics: means, standard deviation. A P value lower than 0.05 was considered significant. Groups were compared for different variables in single time points using a t-test or Mann-Whitney U-test, depending on the distribution. Multiple test correction was not performed.

Microsoft Excel 8.6 (Microsoft, Redmond, Washington, USA) and SPSS for Windows (version 16.1; SPSS Inc, Chicago, Illinois, USA) were used for the analysis.

## Results

### Intraocular pressure and hypotony

**Fig 1:**
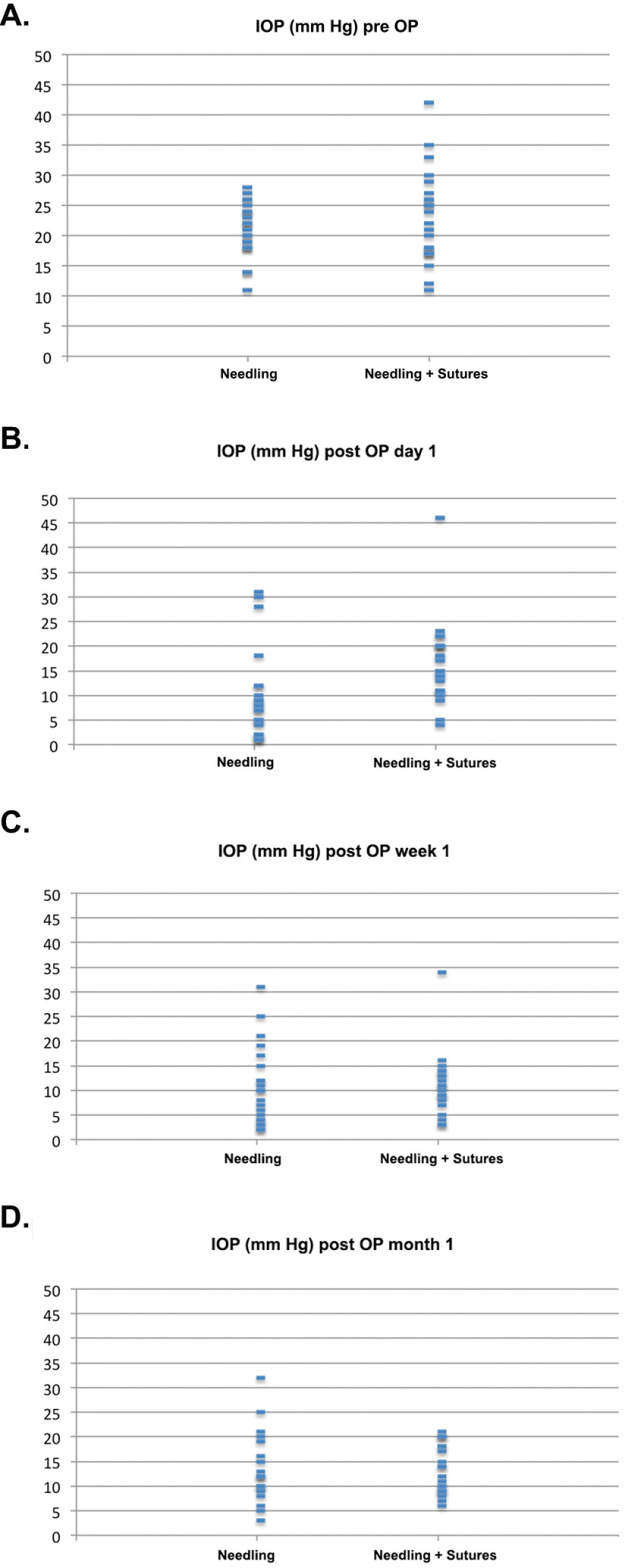
Scattergram of the intraocular pressure (IOP) of both study groups preoperatively (A), one day (B), one week (C) and one month (D) after the bleb revision.

After the initial trabeculectomy and prior to the revision surgery, the mean intraocular pressure (IOP) did not differ significantly between (P = 0.59) the two groups (Table 2, Fig 1A). IOP was 22.96 ± 7.57 mmHg in group 1 (needling procedure) and 21.09 ± 4.37 mmHg in group 2 (needling plus transconjunctival scleral flap sutures). As all patients had a dysfunctional bleb and high intraocular pressure, most needed glaucoma medication during the period before the revision: twenty-one patients in group 1 received on average 0.71 ± 0.90 drugs, while twenty patients in group 2 received on average 0.8 ± 0.95 drugs (P = 0.8).

**Fig 2:**
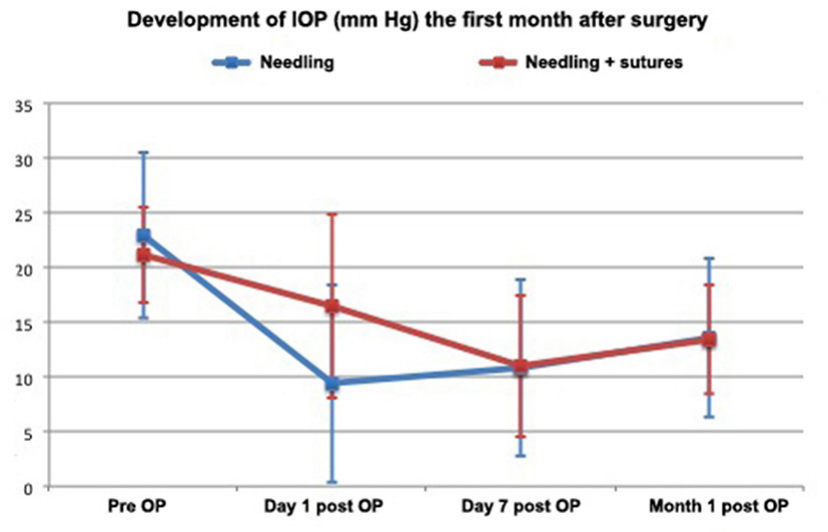
Development of IOP over time (preoperative till 1 month after surgery) in the groups in the study.

The mean IOP on the first postoperative day of patients who received the sole needling procedure was 9.43 ± 9.05 mmHg (Table 2, Figs 1B and 2). In ten cases, IOP was found to be 5 mmHg or lower (ocular hypotony). During the early postoperative period, the IOP increased again and reached 10.81 ± 8.03 mmHg (7 patients with hypotony) after one week (Table 2, Figs 1C and 2) and 13.58 ± 7.24 mm Hg (2 patients with hypotony) after one month (Table 2, Figs 1D and 2).

The mean IOP of patients receiving additional transconjunctival scleral flap sutures to the needling procedure was 16.43 ± 8.35 mmHg on the first postoperative day (significantly higher in comparison to group 1, P = 0.001). Only two patients were found with ocular hypotony (Table 2, Figs 1B and 2). The following time IOP decreased further to 11.00 ± 6.50 mmHg (4 patients with hypotony) at the end of week one (no difference from group 1, P = 0.61) (Table 2, Figs 1C and 2) and to 13.40 ± 4.93 mm Hg (no patients with hypotony) at the end of the first month (Table 2, Figs 1D and 2) (no difference from group 1, P = 0.9).

All patients of both groups received no glaucoma medication in the first month after revision.

### Number of sutures/Postoperative Management/Complications

Because of an intraoperative estimation of a very high bleb filtration and thus of a very low IOP, one to three sutures were placed transconjunctival on the scleral flap of the patients of group 2. The majority of patients (13 out of 23; 57%) received one suture, 8 patients received two sutures, and two patients received three sutures.

Postoperatively, ten patients showed spontaneously satisfactory IOP development without the need for any further manipulation on the transconjunctival scleral flap sutures. Partial removal of the sutures was performed in 13 patients. Of these, two patients developed hypotony after suture removal, requiring a further intervention with new sutures placed.

Patients of the needling group received 3.5 ± 2.72 subconjunctival injections of 5-FU, while these of the needling plus scleral sutures group received an average of 5.7 ± 2.67 injections (P = 0.008). The situation of the cornea, however, was similar between the two groups (three and two patients with corneal erosion respectively, P = 0.64). The hospital stay lasted 5.3±1.61 days in group 1 and 5.83 ± 1.72 in group 2 (P = 0.232) (Table 3).

**Table 3:** Treatment and management of patients of both groups in the study after the bleb revision related to the need of antimetabolites and the time spent in hospital.

Post-OP Management	Needling Group	Needling + Sutures	P Value
Time in hospital (days)	5.3 ± 1.61	5.83 ± 1.72	0.23
5 FU (injections)	3.5 ± 2.72	5.7 ± 2.67	**0.008**

Both procedures proved overall to have a similarly low rate of complications (Table 4). However, one patient in group 1 developed a choroidal hemorrhage (4.3%) and five patients showed choroidal effusion (21.7%). In group 2, no choroidal hemorrhage was observed, two patients had choroidal effusion (8.7%) and one patient had a bleb leakage (4.3%).

**Table 4:** Number of complications: corneal erosion, bleb leakage, choroidal effusion, and subchoroidal hemorrhage.

Complications	Needling	Needling + Sutures	P Value
Corneal Erosion	3 (13%)	2 (8.7%)	0.64
Bleb Leakage	0	1 (4.3%)	0.31
Choroidal Effusion	5 (22%)	1 (4.3%)	0.08
Choroidal Hemorrhage	1 (4.3%)	0	0.31

## Discussion

Our study offers several interesting findings related to the surgical management of encapsulated blebs. First of all, the standard method of needling is a very successful approach for the rehabilitation of aqueous outflow in non-functional encapsulated blebs. Second, the early postoperative period is accompanied by low IOPs, which have the potential to cause known complications, such as anterior chamber swallowing, choroidal effusion or subchoroidal hemorrhage possibly leading to severe visual impairment. Third, it was possible to achieve a smooth and adjustable IOP reduction avoiding early ocular hypotony by performing additional transconjunctival sutures on the scleral flap. Fourth, we showed that the additional use of transconjunctival scleral flap sutures during the standard needling procedure leads to bleb function and intraocular pressure control that is similar to needling alone by 1 month, with no higher reoccurrence rate of bleb encapsulation and failure.

Ocular hypotony after revision surgery for encapsulated blebs is not uncommon, and can cause adverse effects such as choroidal effusion or hemorrhage with an overall incidence reported of between 15–30% [10, 11]. In our study, complications caused by ocular hypotony were present in 26% of patients who underwent a simple needling procedure, but in only 13% of patients who received additional sutures (P>0.05). We believe that this difference is due to the large difference in IOP between the two groups during the early postoperative phase (P<0.05). After the simple needling revision, the mean IOP was 9.43 ± 9.05 mmHg, with 43% of the patients having an IOP lower than 5 mmHg, while after the combined procedure with scleral sutures, the mean IOP was 16.43± 8.35 mmHg and only two patients (8.7%) had an IOP below 5 mmHg (Fig 1). This means that the probability of early ocular hypotony, defined as an IOP of 5 mmHg or less, was five times lower in the group which received the new, modified procedure. The probability of complications related to hypotony was then only half of that in the simple revision group.

Furthermore, it should be noted that additional sutures were placed if the IOP during the operation was lower, and for this reason postoperative hypotony seemed more likely. Thus, the group in which it was more likely to observe hypotony in fact presented fewer cases and fewer related complications. Moreover the only case of a choroidal hemorrhage occurred in the group without additional sutures. Consequently, there seems to be a major protective effect of the additional transconjunctival sutures on the scleral flap, as in such cases incidences of hypotony and of its complications are lower. In the study, two patients showed very high filtration after postoperative removal of the transconjunctival scleral flap sutures and went hypotonic. For these patients, it became necessary to place the sutures on the scleral flap once again in a second procedure as a separate surgical intervention in the operation theatre. The fact that in these cases the IOP was subsequently observed at normal levels indicates that this new method indeed is effective, and prevents hypotony.

A weakness of our study is that it is not a prospective randomized study. Rather, it is a case series, and the data were collected after the method was developed. The number of patients was far too small to show a statistically significant difference in number of cases with hypotony or expulsive hemorrhage. However, the group with the higher risk had fewer complications after this procedure, which is very promising.

On the other hand, one possible challenge of the modified procedure is postoperative management. In order to achieve the target IOP, more manipulations were necessary, making the patients’ care more demanding. In some cases, the IOP remained high after the combined procedure. There were 13 subjects with an IOP over 15 mmHg (one had an IOP of 46 mmHg) on the first postoperative day. A higher IOP in the first postoperative time was our target. In the following days, the transconjunctival scleral flap sutures advance through the conjunctiva and become looser in the subconjunctival space. This causes a further, automatic drop of the IOP to the desired levels. In some cases, however, the sutures may have been too tight, and this smooth drop of the IOP did not occur alone. In these subjects, removal of one or more of our transconjunctival scleral flap sutures was necessary. This could be done easily on the slit lamp with scissors and forceps if the suture was reachable over the conjunctiva, or by argon laser if the suture was already in the subconjunctival space. There was a need for more 5-FU after the combined procedure, as this was decided after taking into consideration the appearance and function of the bleb. This was to be expected and can be explained by the fact that the additional sutures produce more scarring, activate fibrosis and vascularization, and bring the conjunctiva and the tenon’s capsule closer to each other. However, under the appropriate management there were no higher failure rates of the combined procedure in comparison to the sole needling procedure in the first postoperative month. Bleb leakage occurred at the site of entrance and exit of the additional sutures; however, this is in general temporary, as the sutures move deeper under the conjunctiva, which typically then heals itself without problems.

We conclude that our rationale for the development of the needling procedure with additional scleral sutures was sound: this technique is effective in significantly reducing the probability of postoperative early ocular hypotony and its complications. We suggest its use in cases in which hypotony is more likely to occur. We also suggest using this technique in patients with an increased risk of expulsive hemorrhages, such as those with myopia, high preoperative IOPs or who have had previous surgery.

## Supporting Information

S1 File Intraocular Pressure. IOP of both study groups preoperatively, one day, one week and one month after the bleb revision. Presented are also the mean and the standard deviation of each group.
(XLSX)

## References

[1] Errico D, Scrimieri F, Riccardi R, Fedeli R, Iarossi G. Trabeculectomy with double low dose of mitomycin C—two years of follow-up. Clinical ophthalmology. 2011;5:1679–86. 10.2147/OPTH.S25611 22205828 PMC3245191

[2] Occleston NL, Daniels JT, Tarnuzzer RW, Sethi KK, Alexander RA, Bhattacharya SS, et al Single exposures to antiproliferatives: long-term effects on ocular fibroblast wound-healing behavior. Investigative ophthalmology & visual science. 1997;38(10):1998–2007. . 9331263

[3] Green E, Wilkins M, Bunce C, Wormald R. 5-Fluorouracil for glaucoma surgery. The Cochrane database of systematic reviews. 2014;2:CD001132 10.1002/14651858.CD001132.pub2 . 24554410 PMC10558100

[4] De Fendi LI, Arruda GV, Scott IU, Paula JS. Mitomycin C versus 5-fluorouracil as an adjunctive treatment for trabeculectomy: a meta-analysis of randomized clinical trials. Clinical & experimental ophthalmology. 2013;41(8):798–806. . 24308066 10.1111/ceo.12097

[5] Azuara-Blanco A, Katz LJ. Dysfunctional filtering blebs. Survey of ophthalmology. 1998;43(2):93–126. . 9763136 10.1016/s0039-6257(98)00025-3

[6] Sherwood MB, Spaeth GL, Simmons ST, Nichols DA, Walsh AM, Steinmann WC, et al Cysts of Tenon's capsule following filtration surgery. Medical management. Archives of ophthalmology. 1987;105(11):1517–21. . 3675283 10.1001/archopht.1987.01060110063032

[7] Koukkoulli A, Musa F, Anand N. Long-term outcomes of needle revision of failing deep sclerectomy blebs. Graefe's archive for clinical and experimental ophthalmology = Albrecht von Graefes Archiv fur klinische und experimentelle Ophthalmologie. 2015;253(1):99–106. 10.1007/s00417-014-2810-4 . 25303883

[8] Anand N, Khan A. Long-term outcomes of needle revision of trabeculectomy blebs with mitomycin C and 5-fluorouracil: a comparative safety and efficacy report. Journal of glaucoma. 2009;18(7):513–20. 10.1097/IJG.0b013e3181911271 . 19223788

[9] Rotchford AP, King AJ. Needling revision of trabeculectomies bleb morphology and long-term survival. Ophthalmology. 2008;115(7):1148–53 e4. 10.1016/j.ophtha.2007.10.023 . 18082890

[10] Maestrini HA, Cronemberger S, Matoso HD, Reis JR, Merula RV, Filho AD, et al Late needling of flat filtering blebs with adjunctive mitomycin C: efficacy and safety for the corneal endothelium. Ophthalmology. 2011;118(4):755–62. 10.1016/j.ophtha.2010.08.020 . 21055818

[11] Shafi F, Agrawal P, Holder R, Sung V. Bleb needling with subconjunctival injection of sodium hyaluronate 1.4%: 1-year outcomes. Canadian journal of ophthalmology Journal canadien d'ophtalmologie. 2011;46(6):537–42. 10.1016/j.jcjo.2011.09.005 . 22153643

[12] Rulli E, Biagioli E, Riva I, Gambirasio G, De Simone I, Floriani I, et al Efficacy and safety of trabeculectomy vs nonpenetrating surgical procedures: a systematic review and meta-analysis. JAMA ophthalmology. 2013;131(12):1573–82. 10.1001/jamaophthalmol.2013.5059 . 24158640

[13] Schwenn O, Kersten I, Dick HB, Muller H, Pfeiffer N. Effects of early postfiltration ocular hypotony on visual acuity, long-term intraocular pressure control, and posterior segment morphology. Journal of glaucoma. 2001;10(2):85–8. . 11316101 10.1097/00061198-200104000-00003

[14] Nuyts RM, Greve EL, Geijssen HC, Langerhorst CT. Treatment of hypotonous maculopathy after trabeculectomy with mitomycin C. American journal of ophthalmology. 1994;118(3):322–31. . 8085589 10.1016/s0002-9394(14)72956-3

[15] Dinah C, Bhachech B, Ghosh G. Long-term success with autologous blood injection for leaking trabeculectomy blebs. The British journal of ophthalmology. 2010;94(3):392–3. 10.1136/bjo.2009.164160 . 20215386

[16] Cohen SM, Flynn HW Jr., Palmberg PF, Gass JD, Grajewski AL, Parrish RK 2nd. Treatment of hypotony maculopathy after trabeculectomy. Ophthalmic surgery and lasers. 1995;26(5):435–41. . 8963858

[17] Radhakrishnan S, Quigley HA, Jampel HD, Friedman DS, Ahmad SI, Congdon NG, et al Outcomes of surgical bleb revision for complications of trabeculectomy. Ophthalmology. 2009;116(9):1713–8. 10.1016/j.ophtha.2009.04.003 . 19643490

[18] Kawai M, Nakabayashi S, Shimizu K, Hanada K, Yoshida A. Autologous Transplantation of a Free Tenon's Graft for Repairing Excessive Bleb Leakage after Trabeculectomy: A Case Report. Case reports in ophthalmology. 2014;5(3):297–301. 10.1159/000368159 25408669 PMC4224260

[19] Eha J, Hoffmann EM, Pfeiffer N. Long-term results after transconjunctival resuturing of the scleral flap in hypotony following trabeculectomy. American journal of ophthalmology. 2013;155(5):864–9. 10.1016/j.ajo.2012.12.004 . 23394904

[20] Letartre L, Basheikh A, Anctil JL, Des Marchais B, Goyette A, Kasner OP, et al Transconjunctival suturing of the scleral flap for overfiltration with hypotony maculopathy after trabeculectomy. Canadian journal of ophthalmology Journal canadien d'ophtalmologie. 2009;44(5):567–70. 10.3129/i09-123 . 19789593

